# Baoyuan Jiedu decoction alleviating cancer cachexia–Induced muscle atrophy by regulating muscle mitochondrial function in *Apc* ^*Min/+*^ mice

**DOI:** 10.3389/fphar.2022.914597

**Published:** 2022-08-19

**Authors:** Beiying Zhang, Qianyu Bi, Shengqi Huang, Siyuan Lv, Xin Zong, Mengran Wang, Xuming Ji

**Affiliations:** ^1^ School of Basic Medical Science, Zhejiang Chinese Medical University, Hangzhou, China; ^2^ Weifang Nursing Vocational College, Weifang, China; ^3^ Department of Pediatrics, Affiliated Hospital of Shandong University of Traditional Chinese Medicine, Jinan, China; ^4^ Academy of Chinese Medical Science, Zhejiang Chinese Medical University, Hangzhou, China

**Keywords:** cancer cachexia, muscle atrophy, Baoyuan Jiedu decoction, mitochondrial function, *APC* ^*Min/+*^ mice

## Abstract

Cancer cachexia is a complex syndrome that leads to an ongoing loss of skeletal muscle mass in many malignant tumors. Our previous studies have evaluated the effectiveness of Baoyuan Jiedu decoction (BJD) in alleviating cancer-induced muscle atrophy. However, the mechanisms of BJD regulating muscle atrophy could not be fully understood. Therefore, we further investigated the mechanisms of BJD mitigating muscle atrophy both in an *Apc* ^*Min/+*^ mouse model and the Lewis-conditioned medium–induced C2C12 myotube atrophy model. We confirmed the quality of BJD extracts by HPLC. In an *In vivo* study, body weight loss and muscle atrophy were alleviated with BJD treatment. GO analysis suggested that ATP metabolism and mitochondria were involved. The results of the electron microscope show that BJD treatment may have a healing effect on mitochondrial structure. Moreover, ATP content and mitochondrial numbers were improved with BJD treatment. Furthermore, both *in vivo* and *in vitro*, we demonstrated that the BJD treatment could improve mitochondrial function owing to the increased number of mitochondria, balanced dynamic, and regulation of the electron transport chain according to the protein and mRNA expressions. In addition, oxidative stress caused by mitochondrial dysfunction was ameliorated by BJD treatment in *Apc* ^*Min/+*^ mice. Consequently, our study provides proof for BJD treatment alleviating cancer cachexia–induced muscle atrophy by modulating mitochondrial function in *Apc* ^*Min/+*^ mice.

## Introduction

Cancer-associated cachexia is a multifaceted, irreversible, and multiorgan syndrome that occurs in most cancer patients and results in 30% of deaths among them. It is characterized by an ongoing loss of body weight with specific loss of skeletal muscle and progressive functional impairment (Fearon et al., 2011; Martin, 2016; Schmidt et al., 2018; Siff et al., 2021). Muscle atrophy is the main cause of the loss of body weight in cancer cachexia (Argiles et al., 2014a). However, there is no effective drug to reverse muscle consumption at present. Therefore, it is crucial to find effective approaches to mitigate muscle atrophy in cancer cachexia.

An imbalance of energy requirements and energy uptake weakens skeletal muscle strength in cancer cachexia (Rohm et al., 2019). Mitochondria play an important role in the synthesis of ATP in skeletal muscle and hence as a cellular regulator of muscle atrophy in cancer-induced cachexia (Carson et al., 2016). Mitochondria maintain capacity through regulating the generation, fusion, fission, and autophagy to fulfill the demands of energy metabolism (Yan et al., 2012). Studies have illustrated that cancer-induced cachexia leads to muscle atrophy which is caused in part by mitochondrial dysfunction (de Castro et al., 2019). Furthermore, oxidative stress caused by mitochondrial dysfunction also results in muscle atrophy (Powers et al., 2012). Therefore, improving mitochondrial function is one of the strategies for treating muscle atrophy in cancer cachexia.

The antitumor curative effects of traditional Chinese medicine (TCM) have been widely acknowledged (So et al., 2019; Xu et al., 2020; Ma et al., 2021). Baoyuan Jiedu decoction (BJD) is a classic traditional Chinese herbal formula for treating cancer cachexia. Our prior studies have evaluated the efficacy of BJD in relieving muscle atrophy in Lewis lung carcinoma–induced cancer cachexia mice and C26 colorectal tumor–bearing mice by downregulating the expressions of atrogin-1 and MuRF-1 (Zhang et al., 2017; Wang et al., 2020). Furthermore, it has been proved that BJD could suppress the expressions of atrogin-1 and MuRF-1 via inhibiting the ubiquitin–proteasome pathway in the Lewis-conditioned medium (LCM)–induced C2C12 myotube atrophy model (Zhang et al., 2018). In addition, we found that BJD could alleviate cancer-induced myotube atrophy by improving mitochondrial dynamics through the p38 MAPK/PGC-1α signaling pathway both *in vivo* and *in vitro* (Wang et al., 2020). Otherwise, we used the *Apc* ^*Min/+*^ mouse model, a spontaneous intestinal tumorigenesis model, which likewise demonstrated that BJD could prevent muscle atrophy by downregulating the expressions of atrogin-1 and MuRF-1, and mitochondrial uncoupling was involved (Zong et al., 2019).

In this study, we further elucidated the mechanisms underlying the impact of BJD alleviating cancer cachexia muscle atrophy on an *Apc* ^*Min/+*^ mouse model and the LCM-induced C2C12 myotube atrophy model by modulating mitochondrial function.

## Materials and methods

### Preparation of the extracts for BJD and HPLC conditions

The composition of BJD is *Panax ginseng* C.A.Mey., *Aconitum carmichaelii* Debx., *Astragalus mongholicus* Bunge., *Angelica sinensis* (Oliv.) Diels., *Lonicera japonica* Thunb., and *Glycyrrhiza uralensis* Fisch. ex DC. in a ratio of 9:9:18:15:12:6 (9.0, 9.0, 18, 15, 12, and 6.0 g). All herbs were purchased from Zhonglu Hospital (Shandong, China) and identified by the professors from the Department of Pharmacy, Shandong University of Traditional Chinese Medicine (Shandong, China). The method of preparing extracts of BJD was consistent with the study we published previously (Wang et al., 2020), as hot water extracts from the six crude herbs that concentrated to 1.15 g drug/ml.

According to Supplementary Table S1, chlorogenic acid, ferulic acid, and aconitine were qualitatively and quantitatively analyzed by HPLC (Shimadzu, Japan, LC-2010A) to ensure the quality of BJD extracts. The information on standard samples is given in Supplementary Table S2.

### Mice

First, 30 males of the *Apc* ^*Min/+*^ cachexia mouse model at the age of 14 weeks and 10 males of the C57BL/6 J mouse model with the same age and same genetic background were purchased from the Institute of Model Zoology, Nanjing University (Jiangsu, China). The Experimental Animal Center of Shandong University of Traditional Chinese Medicine (Shandong, China) provided SPF-grade animal experimental environment and daily support, and our experiment was approved by the Animal Ethical and Welfare Committee of Shandong University of Traditional Chinese Medicine (ID: SDUTCM201805311223). Mice were divided as shown in Figure 2A. Thirty *Apc* ^*Min/+*^ mice were divided equally into three groups, as the BJD group, the megestrol acetate group (MA), and the model group. C57BL/6 J mice were used as a blank control group (normal group). According to our previous study (Wang et al., 2020), the dose of the BJD group was 23 g/kgd by gavage. The MA group was intraperitoneally injected with 24 mg/kgd of megestrol acetate (Ape×Bio, United States, Cat# B1377). The model group and the normal group were given an equal dose of saline by gavage once per day. They were all administered for 12 weeks continuously and weighed every day. Finally, after drawing the blood from the eyeball, mice were sacrificed by dislocation of the cervical to get the gastrocnemius muscle. Also, the grip strength of mice was recorded that morning. The front paws of the mice were covered with infusion tubes, rendering them incapable of grasping. Forelimb grip force was then measured by lifting the mouse tail and gently and steadily pulling it back. The value was recorded when both hind limbs released the grip rod at the same moment. The test was repeated 4–6 times to ascertain the maximal grip strength.

### H&E staining and electron microscopy

Gastrocnemius tissues were rinsed with 0.9% saline and fixed with 4% paraformaldehyde for 24 h. Then, tissues were embedded in paraffin (JB-P5, Junjiedianzi, China) and cut into slices (RM 2016, Leica, Germany). After the gradient was dehydrated to drain xylene, tissues were stained in hematoxylin for 3–5 min. They were washed in running tap water, dipped in 1% acid alcohol for differentiation, washed again, and stained in 1% eosin. Before being sealed with neutral balsam, tissues were treated with alcohols and xylene. In the end, tissues were placed on microscope slides for H&E examination (Nikon Eclipse E100, Nikon, Japan).

Tissues were fixed with 2.5% glutaraldehyde for 2 h immediately at 4°C, rinsed in PBS, and then fixed with 1% osmium tetroxide for 2 h at room temperature. Then, they were rinsed, dried, infiltrated, and embedded for 48 h at 60°C at last and cut into slices. After staining, the samples were observed and photographed by a transmission electron microscope (JEM-1400, JEOL, Japan).

### Transcriptome sequencing

Total RNA was extracted from the gastrocnemius muscle using the mirVana miRNA Isolation Kit (Ambion, United States), following the manufacturer’s protocol. The libraries were constructed using the TruSeq Stranded mRNA LTSample Prep Kit (Illumina, San Diego, CA, United States). Then, libraries whose RNA Integrity Number (RIN) ≥ 7 were subjected to being sequenced on the Illumina sequencing platform (HiSeqTM 2500) after being evaluated by Agilent 2100 Bioanalyzer (Agilent Technologies, Santa Clara, CA, United States). To get clean reads, raw reads were filtered through the NGS QC Toolkit.

We compared the resulting clean reads to the mice reference genome using Bowtie2. Analysis to categorize significant differentially expressed genes was performed by DESeq software. Thus, we identified genes with a fold change (FC) > 1.5 and a *p*-value <0.05 in comparison as DEGs. The DEGs were shown in the heatmap and subsequently subjected to enrichment analysis of Gene Ontology (GO) analysis and Kyoto Encyclopedia of Genes and Genomes (KEGG) analysis.

### Colorimetric method

We used the ATP Assay Kit (Ruifan, China) to detect the ATP content in the gastrocnemius muscle. Reagents were added in turn to the gastrocnemius muscle in strict accordance with the manufacturer’s instructions and fully mixed in a water bath at 37°C for 30 min. Then, a color-developing agent was added proportionally to the water bath at 37°C for 20 min. The spectrophotometer was preheated (OD1000+, One drop, China) for 30 min, and then it was set to zero with distilled water; the absorbance value of each tube at the wavelength of 700 nm was measured, and the ATP content was calculated according to the formula.

### ELISA

Blood was separated into serum using a centrifugal machine at 1,000 r/min, 20 min (Centrifuge 5415D, Eppendorf, German). All factors were detected by enzyme-linked immunosorbent assay (ELISA) kits (Meimian, China). According to the manufacturer, we set standard wells and testing sample wells. After incubation for 60 min at 37°C, the samples were washed five times and patted dry. Chromogen solution A and chromogen solution B were added in turn, and then the light preservation was evaded for 15 min at 37°C. The Blank well was taken as zero, and absorbance was recorded at 450 nm after adding the stop solution. The sample density with the sample OD value was calculated according to the linear equation with the standard density and the OD value.

### Cell cultivation conditions

The Lewis cells and C2C12 myoblast were purchased from the Shanghai Institutes for Biological Sciences of the Chinese Academy of Sciences (Shanghai, China, Cat#TCM7, Cat#GNM26). DMEM/high glucose medium (Invitrogen, United States) supplemented with 10% fetal bovine serum (Invitrogen, United States), 100 U/mL penicillin, and 100 μg/ml streptomycin composed the medium for the growth. DMEM/high glucose medium (Invitrogen, United States) supplemented with 2% horse serum (Invitrogen, United States), 100 U/mL penicillin, and 100 μg/ml streptomycin composed the medium for the differentiation. According to our previous study (Wang et al., 2020), first, Lewis cells were cultured based on the aforementioned conditions for 2 days at 37°C in 5% CO_2_. Then, the conditioned medium was collected and filtered through a 0.22-µm membrane. In the end, the conditioned medium was mixed with a fresh differentiation medium in a ratio of 1:2, which was named Lewis-cell–conditioned medium (LCM).

C2C12 cells were seeded in 10-cm Petri dishes and cultured in the growth medium. When the cell fusion reached about 90%, the medium was changed to the differentiation medium to induce myotube formation. After 72 h, when myotube was formed, the cells were randomly divided into three groups (*n* = 3): normal group, model group, and BJD group. The normal group was cultured in the fresh differentiation medium, while the model group and BJD group were cultured in LCM. Additionally, BJD decoction medicated serum (10%) was added to the BJD group. BJD decoction–medicated serum was prepared according to our previous study (Zhang et al., 2017). In brief, rats were given BJD decoction (41 g/kg) by gavage for 3 days. Before blood collection, rats were fasted for 12 h after the last administration and then given BJD decoction 1 d dosage. Then, blood was inactivated at 63.5°C and made into freeze-dried powder for subsequent study. All the cell groups were cultured for 72 h at 37°C in 5% CO_2_ for subsequent studies. Morphological performance was observed and transverse diameters of myotubes were processed by ImageJ software.

### mtDNA extraction and qPCR analysis

mtDNA was isolated using the TGuide Cell/Tissue Genomic DNA Extraction Kit (Tiangen, Beijing, China). Trizol reagent (Invitrogen, United States) was used to extract the total RNAs from the gastrocnemius muscle. qPCR was performed by applying TIANScript RT KIT (Tiangen, China, Cat# KR104-02) and SYBR Green (Tiangen, China, Cat# FP205) to detect the expression of mRNA. *ß*-actin was used as an internal gene to normalize the gene expression, while for mtDNA, *ß*-globin was used. The calculation of relative quantification was based on the 2^−△△CT^ method. All of the primers are presented in Supplementary Table S3 (Sangon Biotech, Shanghai, China).

### Western blotting

Tissues and cells were washed twice in 1 × PBS, then lysed (Beyotime, China, P0013 B) for 30 min, centrifuged at 12,000 rpm for 10 min, and the supernatant was collected at the end. The protein concentration was quantified by a BCA assay kit (Thermo Fisher, United States). Samples were extracted and resolved on 10% SDS-PAGE and transferred to PVDF membranes (Millipore, United States). Then, the membranes were blocked in 5% nonfat milk powder diluted in TBS-T for 2 h before incubation with primary antibodies (RabMAbs, Abcam, United States). On the second day, the membranes were washed three times and then incubated with secondary antibodies (HRP-conjugated goat anti-mouse IgG, Boster, China) at 1:3,000 dilution. The blots were visualized with enhanced chemiluminescence, and the intensity was analyzed by ImageJ software. Values were normalized by GAPDH as an internal control, respectively.

### Statistical analysis

Statistical analyses were performed using SPSS 26.0 (SPSS Inc., United States), and data were presented as means ± SD. Differences between different groups were compared using one-way ANOVA with Fisher’s LSD test. *p* < 0.05 was considered a statistically significant difference.

## Results

### Quality evaluation of BJD extracts

**FIGURE 1:**
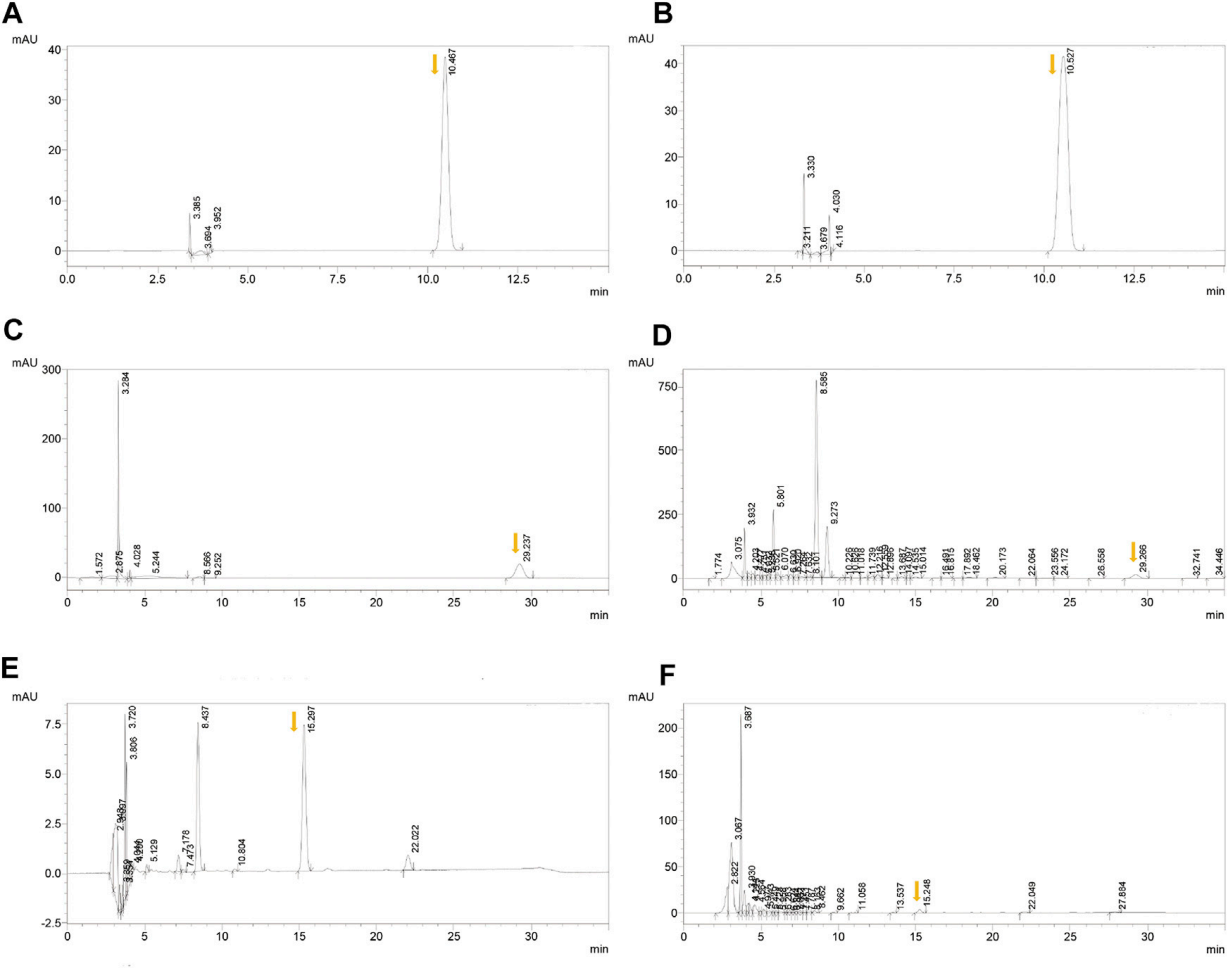
HPLC results of BJD extracts. **(A)** Chlorogenic acid in the standard sample. **(B)** Chlorogenic acid in BJD extracts. **(C)** Ferulic acid in the standard sample. **(D)** Ferulic acid in BJD extracts. **(E)** Aconitine in the standard sample. **(F)** Aconitine in BJD extracts. The arrows point to the target peak.

Chlorogenic acid, ferulic acid, and aconitine indicated a good linear relationship in the range of 0.06–0.30, 0.025–0.125, and 0.02–0.10 μg, respectively (Table 1 and Figure 1). The results of the quantitative evaluation are shown in Table 2, and it could be concluded that the extracts of BJD were stable.

**TABLE 1:** Linear relationship.

	Linear regression equation of peak areas and sample injection volume (μg)	R-value	Range of linear relationship (μg/ml)	Retention time (min)
Chlorogenic acid	Y = 3E-07x+0.0005	0.9997	0–300	10.536
Ferulic acid	Y = 2E-07x-0.0004	0.9999	0–125	29.231
Aconitine	Y = 8E-07x+0.0003	1	0–100	15.295

**TABLE 2:** Quantitative evaluation.

	Extracts sample 1	Extracts sample 2	Extracts sample 3
Chlorogenic acid (μg/ml)	114.4	114.5	114.3
Ferulic acid (μg/ml)	65.99	66.01	65.89
Aconitine (μg/ml)	14.68	14.6	14.76

### BJD alleviated the body weight loss of *Apc* ^*Min/+*^ mice

**FIGURE 2:**
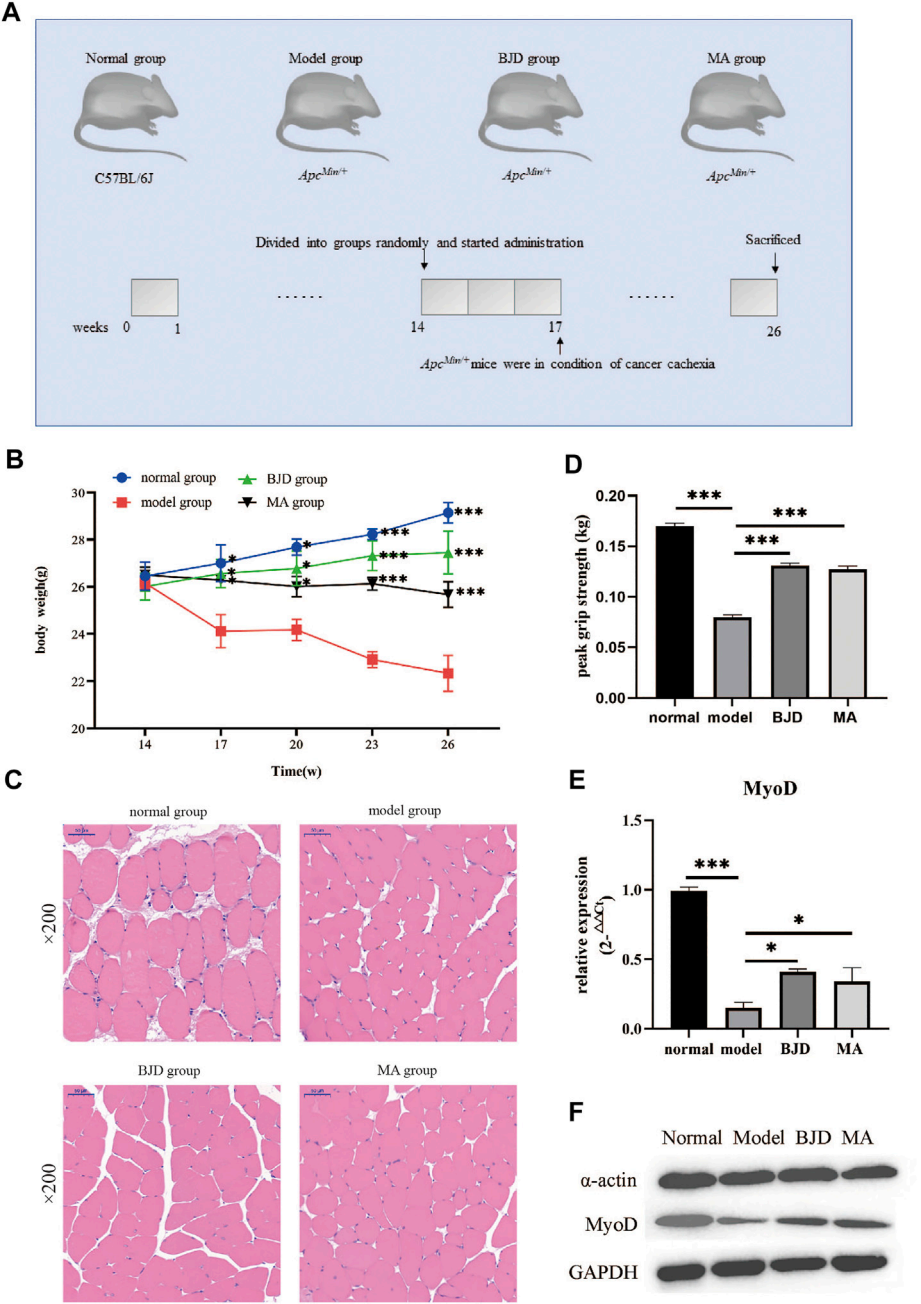
BJD relieved *Apc* ^*Min/+*^ mice muscle atrophy. **(A)** Diagram presenting the mouse experiments. **(B)** The changes in body weight of each group; **(C)** the pictures of the gastrocnemius muscle stained by H&E (×200 magnification; scale bar: 50 µm); **(D)** the changes in grip strength of each group; **(E)** the relative expressions of MyoD detected by qPCR, with *ß*-actin used as an internal gene; and **(F)** the expressions of *a*-actin and MyoD by western blotting, with GAPDH used as a loading control. The data are presented as the mean ± SD. Compared with model group, *p < 0.05, ***p < 0.001.

According to the definition of cancer cachexia, body weight loss could index the severity of cancer cachexia. As shown in Figure 2B, the body weight of mice became significantly different when the mice were 17 weeks old. In the end, BJD significantly alleviated the *Apc* ^*Min/+*^ mice’s body weight loss.

### BJD relieved *Apc* ^*Min/+*^ mice muscle atrophy

The gastrocnemius muscle was stained with HE to examine whether BJD could prevent muscle atrophy (Figure 2C). In addition, the grip strength improved with the BJD treatment (Figure 2D). Similarly, the protein synthesis of muscle was also increased (Figures 2E,F). Accordingly, the results demonstrated that BJD treatment promoted the atrophy of the gastrocnemius muscle compared with the model group.

### BJD affected mitochondrial biology and metabolism in *Apc* ^*Min/+*^ mice

**FIGURE 3:**
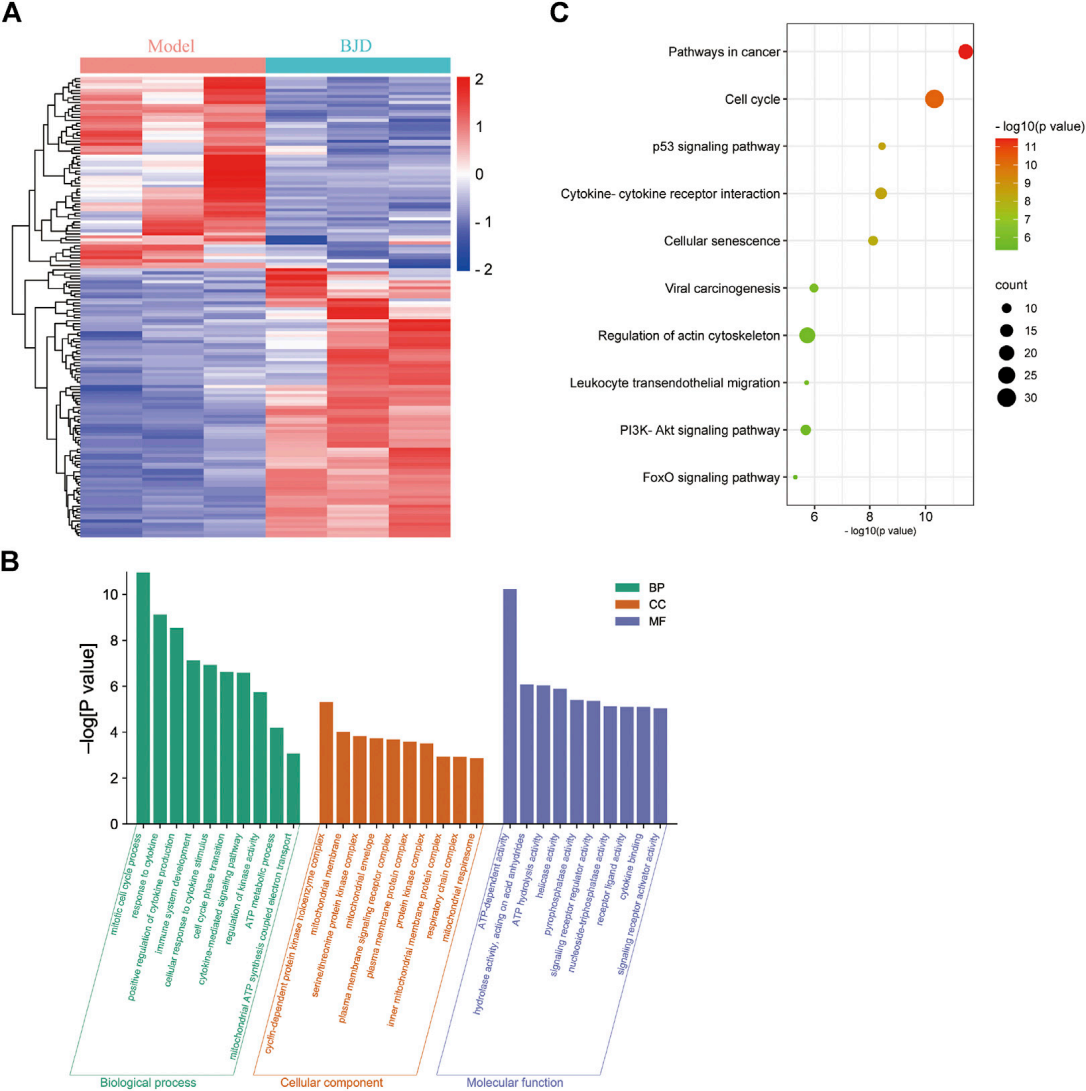
BJD affected ATP metabolism and mitochondria in *Apc* ^*Min/+*^ mice. **(A)** Heatmap of DEGs between the BJD group and the model group; **(B)** GO enrichment analysis of DEGs; and **(C)** KEGG enrichment analysis of DEGs.

Compared with the model group, there were 154 DEGs in the BJD group, including 90 upregulated and 64 downregulated DEGs (Figure 3A). Then, we performed enrichment analysis (Figures 3B,C). Notably, many top GO terms were centered on mitochondrial biology and metabolism, including ATP metabolic process, mitochondrial ATP synthesis coupled electron transport, mitochondrial membrane, and ATP-dependent activity, which revealed that mitochondrial biology and metabolism were affected in the BJD treatment of BJD treatment in *Apc* ^*Min/+*^ mice. Moreover, the KEGG enrichment showed that its mechanisms may be related to the p53 signaling pathway, PI3K–Akt signaling pathway, or FoxO signaling pathway.

### BJD regulated the mitochondrial function

**FIGURE 4:**
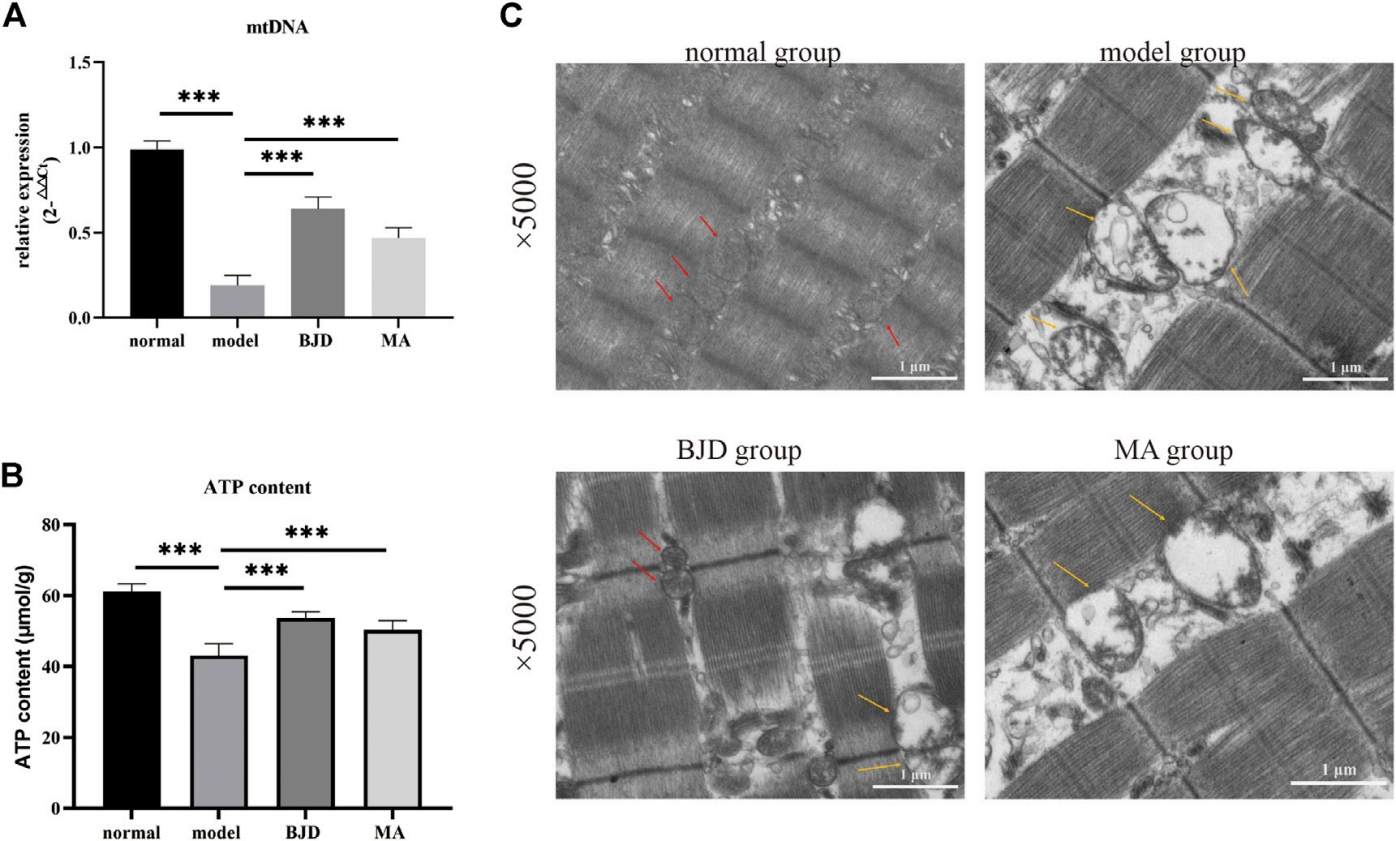
BJD promoted mitochondrial function in *Apc* ^*Min/+*^ mice. **(A)** mtDNA was detected by qPCR, with *ß*-actin used as an internal gene; **(B)** ATP content was detected using the colorimetric method; and **(C)** mitochondrial morphology using transmission electron microscopy (×5.0 k). Scale bars represent 1 μm. The red arrow points to functional mitochondria; the yellow arrow points to vacuolar and dysfunctional mitochondria. The data are presented as the mean ± SD. ***p < 0.001.

**FIGURE 5:**
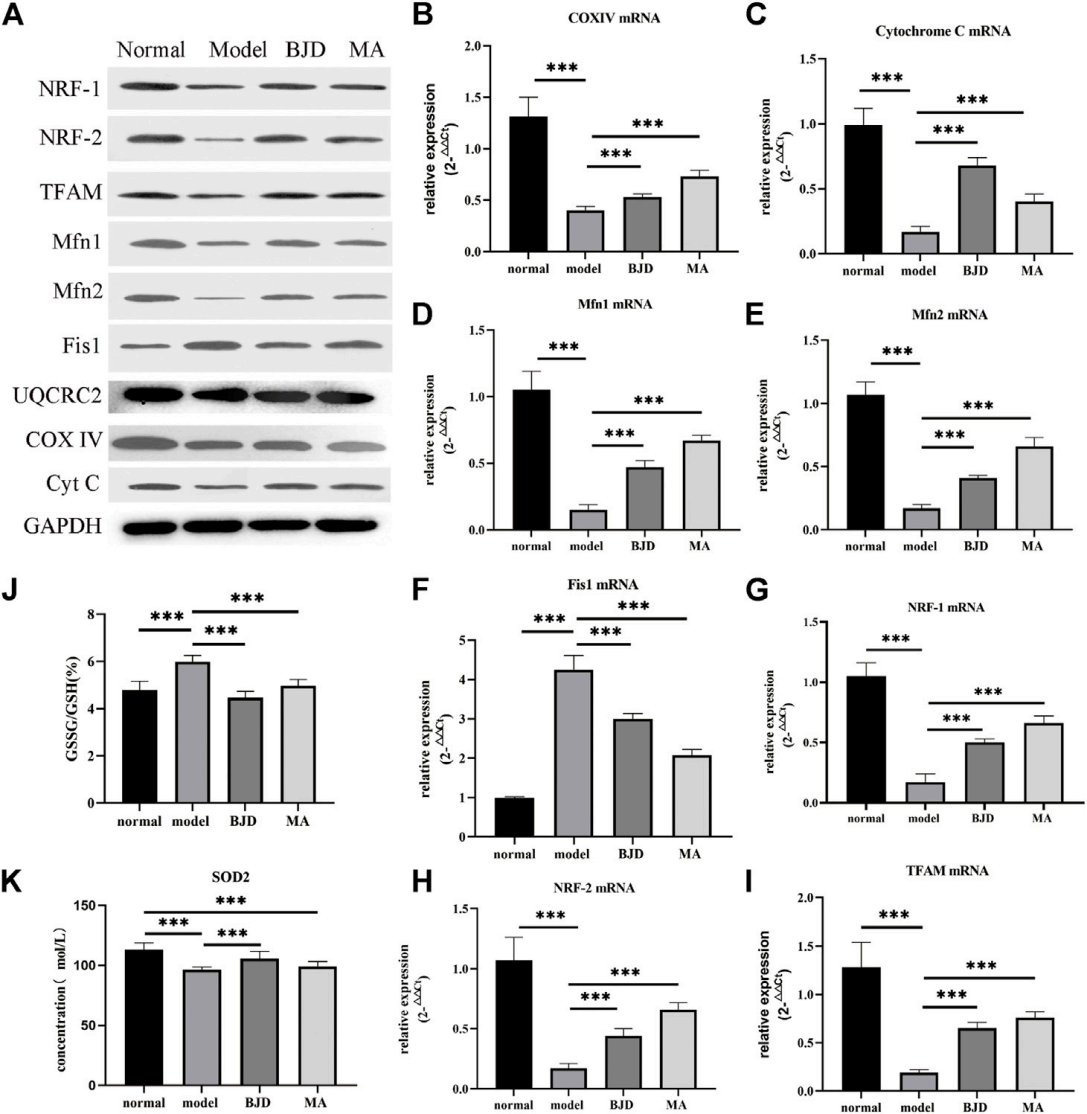
BJD regulated mitochondrial function in *Apc* ^*Min/+*^ mice. **(A)** The relative expressions of proteins related to mitochondrial function were detected by western blotting *in vivo*, with GAPDH used as a loading control. **(B–I)** The relative expressions of genes related to mitochondrial function were detected by qPCR, with *ß*-actin used as an internal gene. **(J,K)** GSSG/GSH and SOD2 were detected by ELISA. The data are presented as the mean ± SD. ***p < 0.05.

ATP content was detected by a colorimetric method, and mtDNA was assessed by qPCR, respectively, showing the effects of BJD treatment on mitochondrial function (Figures 3B, 4A). We further measured the expression levels of the subunits of the mitochondrial electron transport chain (ECT) complexes III and IV. UQCRC2 was the subunit of complex III, and COX IV was the subunit of complex IV, and we also detected cytochrome c to verify the results (Figures 5A–C). Accordingly, mitochondrial function was improved by BJD treatment compared with the model group.

Based on electron microscopy (Figure 4C), as we could see, a large number of the skeletal muscle fibers were damaged and arranged in disorder, and the Z-line was twisted and broken in the model group. Moreover, mitochondria swelled and became round, with lots of vacuolated mitochondria, or even disappeared. While the muscle fibers of the mice in the BJD group were more complete and better arranged in order, the Z-line was neater. Most mitochondria were normal, and the number and fusion of mitochondria increased. Thus, we could conclude that the mitochondrial morphology has changed, suggesting the efficacy of BJD in improving the function of mitochondria.

### BJD improved the generation and dynamic balance of mitochondria

To probe into the mechanism of BJD regulating mitochondrial function, we used western blotting and qPCR to determine the protein and mRNA levels of aiming factors. NRF-1, NRF-2, and TFAM were essential mediators in promoting mitochondrial generation. Fis1 was related to mitochondrial fission. Both Mfn1 and Mfn2 had a connection with mitochondrial fusion. As a result, BJD not only significantly improved the generation of mitochondria but also regulated the dynamic balance of mitochondria compared with the model group (Figures 5A,D–I).

### BJD modulated oxidative stress *in vivo*

Considering one sequence of mitochondrial dysfunction was the formation of oxidative stress, we measured the levels of SOD2 and GSH/GSSG by ELISA (Figures 5J,K). Consequently, the results illustrated that BJD treatment modulated oxidative stress, resulting in the reduction of muscle atrophy.

### BJD mediated mitochondrial function *in vitro*

**FIGURE 6:**
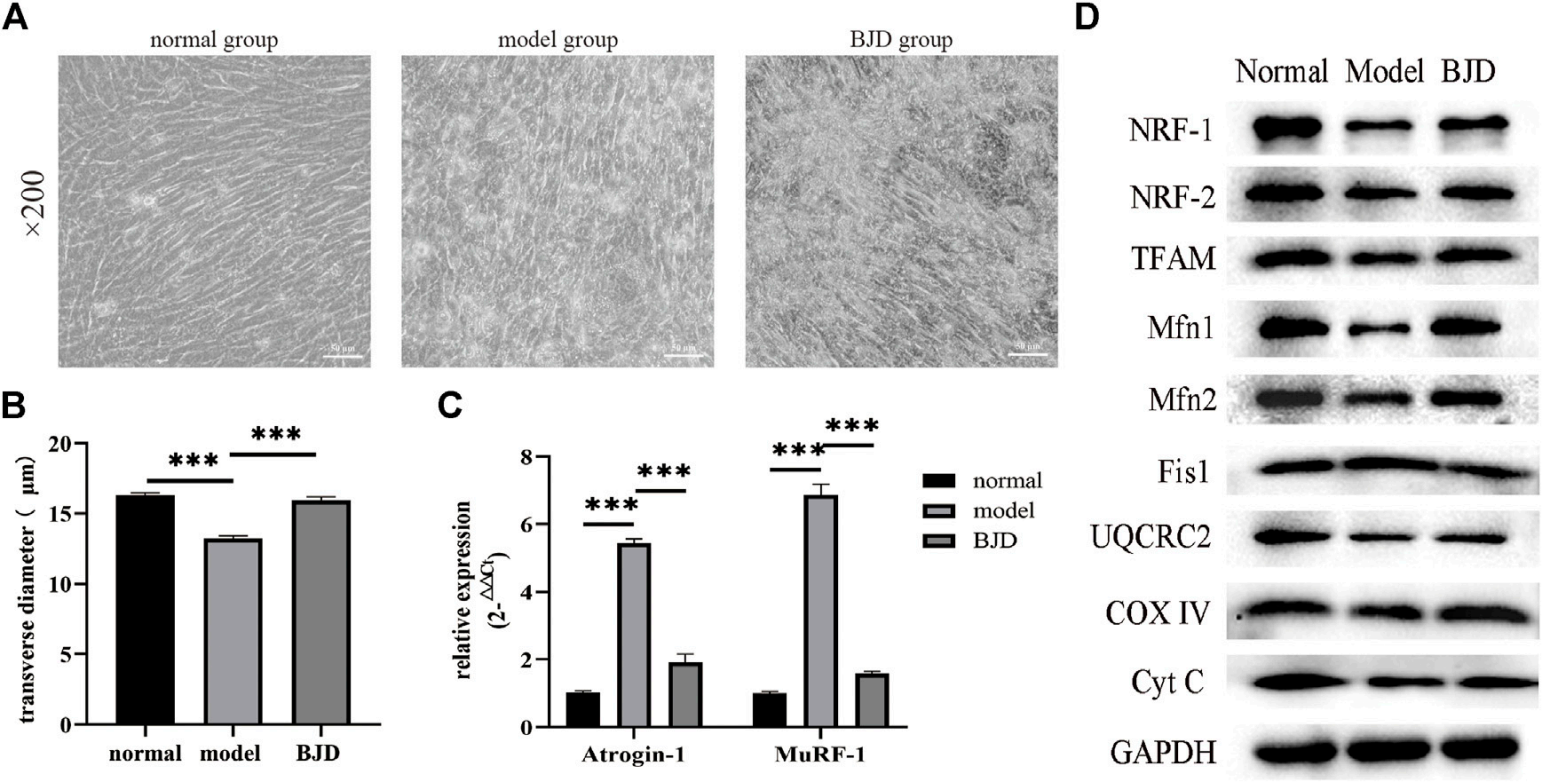
BJD prevented LCM-induced myotube atrophy in C2C12 cells. **(A)** The morphological changes of myotubes in C2C12 cells (×200 magnification; scale bar: 50 µm). **(B)** The transverse diameters of myotubes in C2C12 cells (µm, *n* = 3). **(C)** The relative expressions of atrogin-1 and MuRF-1 were detected by qPCR, with *ß*-actin used as an internal gene. **(D)**The relative expressions of proteins related to mitochondrial function were detected by western blotting *in vitro*, with GAPDH used as a loading control. The data are presented as the mean ± SD. ***p < 0.05.

To further examine whether the effect on BJD preventing muscle atrophy was achieved via mitochondrial function, we elucidated these protein expressions *in vitro* by using the LCM-induced C2C12 myotube atrophy model (Figure 6A). With BJD treatment, myotube atrophy was improved according to the myotube transverse diameter measurement and the expression of muscle-specific atrophy marker proteins (Figures 6B,C). Therefore, to investigate the effect of BJD on mitochondria *in vitro*, we used western blot to detect the mitochondria-related protein expressions. The results showed that BJD treatment mediated mitochondrial function *in vitro* (Figure 6D).

## Discussion

Cancer cachexia is a multifactorial syndrome defined and classified by progressive weight loss due to persistent skeletal muscle atrophy (Fearon et al., 2011), leading to a negative effect on life quality, responsiveness to chemotherapy, system perturbations, inflammation, and survival rate (Argiles et al., 2012; Penet and Bhujwalla, 2015; Baracos et al., 2018; Siddiqui et al., 2020). The pathogenesis of muscle atrophy in cancer cachexia is complicated and not clear so far. Studies have shown that it is connected with inflammation, increased skeletal muscle degradation, muscle protein synthesis disorder, disrupted energy balance, and altered mitochondrial function (Sandri, 2016; Aversa et al., 2017; Baracos et al., 2019; Pin et al., 2019). Both cancer cachexia mice and patients have found dysfunctional mitochondria in skeletal muscle (de Castro et al., 2019; Neyroud et al., 2019). Moreover, it remains an unmet medical request and needs a standard management guideline (Sadeghi et al., 2018).

Cancer cachexia is regarded as a consumptive disease in traditional Chinese medicine. Baoyuan Jiedu decoction is a classic Chinese formula consisting of six herbs, including, *Panax ginseng* C.A.Mey., *Aconitum carmichaelii* Debx., *Astragalus mongholicus* Bunge., *Angelica sinensis* (Oliv.) Diels., *Lonicera japonica* Thunb., and *Glycyrrhiza uralensis* Fisch. ex DC. in a ratio of 9:9:18:15:12:6 (9.0, 9.0, 18, 15, 12, and 6.0 g). We have observed that BJD can effectively reduce the degree of fatigue while also improving the clinical symptoms and the sleep quality of patients with cancer (Wang et al., 2021), and the main components in the BJD extract had previously been identified by UHPLC-Q exactive analysis (Wang et al., 2020). Furthermore, we indicated that BJD can improve mitochondrial function via the p38 MAPK/PGC1α signaling pathway in C2C12 cells (Wang et al., 2020). However, the mechanisms of BJD alleviating muscle atrophy in cancer cachexia remain unclear, and the specific influence on mitochondrial function has not been confirmed. As a result, we used a spontaneous intestinal tumorigenesis model for subsequent research since it exhibits a slow-progressive cachectic phenotype similar to human cancer cachexia (Baltgalvis et al., 2010; Suzuki et al., 2020).

According to the material basis we identified before, we further verified the quality of different batches of BJD extracts (Wang et al., 2020). We applied high-performance liquid chromatography (UPLC) to detect the linear relationships and quantities. The findings revealed good linear relationships and stable quantities of BJD extracts, indicating stability. Importantly, both chlorogenic acid and ferulic acid have obvious antitumor and anti-inflammation efficacy. Chlorogenic acid could treat cancer by influencing cancer cell differentiation, and the content of ATP was decreased after intervention (Huang et al., 2019). Ferulic acid has been reported to have low toxicity, possessing a variety of physiological effects including anti-inflammatory, antioxidant, and anticancer (Yang et al., 2015; Zdunska et al., 2018). Despite the presence of poisonous aconitine in BJD extracts (Chan, 2009), it was found in low concentrations in our study, indicating safety. Thus, giving another kind of material basis of BJD for treating cancer cachexia.

Cancer cachexia is characterized by muscle atrophy, and we have proved that BJD treatment could alleviate muscle atrophy in cancer cachexia. Atrogin-1 and MuRF-1 were two key muscle-specific E3 ubiquitin ligases regarded as the markers of muscle atrophy, and also proved to be related to the mechanisms of BJD improving muscle atrophy in cancer cachexia (Bodine and Baehr, 2014; Zhang et al., 2017; Zhang et al., 2018; Wang et al., 2020). Studies have shown that atrogin-1 and MuRF-1 targeted several myofibrillar proteins for degradation, including *a*-actin within the ubiquitin–proteasome system (Peris-Moreno et al., 2021). Considering that we have previously proved that BJD treatment inhibited the protein expressions of atrogin-1 and MuRF-1 in *Apc* ^*Min/+*^ mice (Supplementary Figure S2), we detected the protein expression of *a*-actin and MyoD in this study (Figures 2E,F). The results suggested that BJD had a possible curative effect on alleviating muscle atrophy in *Apc* ^*Min/+*^ mice.

*Apc* ^*Min/+*^ mice are heterozygous for a point mutation in the *adenomatous polyposis coli* (APC) gene (Supplementary Figure S1), and are widely used to research familial adenomatous polyposis and colorectal tumors (Ren et al., 2019). Therefore, we applied transcriptome sequencing to clarify the mechanisms of BJD ameliorating muscle atrophy in cancer-associated cachexia. GO analysis demonstrated that the DEGs were associated with mitochondrial biology and metabolism (Figure 3B). In addition, we noticed that KEGG analysis showed that regulation of actin cytoskeleton was involved, which was consistent with the results of muscle state–related protein expressions (Figures 2E,F). Collectively, all of these results are highly related to mitochondrial function, which tallies with our previous study and prompts us to focus on mitochondria.

To further dissect the underlying mechanisms of the effects of BJD treatment on alleviating muscle atrophy in *Apc* ^*Min/+*^ mice by regulating mitochondrial function, we confirmed the mitochondrial function based on our prior results. We testified that increased content of ATP in *Apc* ^*Min/+*^ mice indicates the alleviation of energy synthesis with BJD treatment (Figure 4B). Mitochondrial dysfunction is connected to reduced mitochondrial content, decreased ability for mitochondrial synthesis, unbalanced mitochondrial dynamics, altered morphology, decreased activity of the complexes of the ECT, the opening of the mitochondrial permeability transition pore, the formation of oxidative stress, and uncoupling, all of which leads to skeletal muscle atrophy in cancer cachexia (Constantinou et al., 2011; Fermoselle et al., 2013; Argiles et al., 2014b; Argiles et al., 2015; Boengler et al., 2017; van der Ende et al., 2018). Therefore, we observed mitochondrial morphology with the electron microscope, concluding that both mitochondrial morphology and muscle structure had improved under BJD treatment. Mitochondrial biogenesis is required to maintain the content and function of mitochondria in muscle. In addition, mitochondrial DNA (mtDNA) also encodes essential genes for energy production, reflecting the energy metabolism (Wallace, 2005). Accordingly, we detected the expressions of mtDNA, respiratory factor 1 (NRF-1), respiratory factor 2 (NRF-2), and mitochondrial transcription factor A (TFAM), demonstrating the improvement in the generation of mitochondria (Figures 5A,G–I). Mitochondrial dynamics which involve the combination of fission and fusion are critically important for the efficiency of oxidative phosphorylation affecting energy metabolism (Ding et al., 2010; Romanello et al., 2010; van der Ende et al., 2018). Similarly, the dynamics of mitochondria were balanced for the suppression of fission and the augmentation of fusion with BJD treatment (Figures 5A,D–F). The ECT participates in oxidative phosphorylation and produces ATP. Moreover, mtDNA encodes several subunits of mitochondrial complexes (Wallace, 2005). Studies have shown that oxidative stress and the function of complexes III and IV of ETC could be regulated by MIA40 glutathionylation. In addition, complex IV activity shows the strongest association with oxidative capacity in the human skeletal muscle (Larsen et al., 2012). Therefore, we detected the expressions of subunits of complexes III and IV to figure out the activity of the ECT (Figures 5A–C). The expressions of subunits indicated the integrity of complexes was damaged because of cancer cachexia, suggesting that the activity of these two complexes was inhibited and the BJD treatment could promote these changes. Accordingly, the formation of oxidative stress resulting from mitochondrial dysfunction can be modulated by the members of the mitochondrial uncoupling protein (UCP) family. We detected the levels of UPC2 and UCP3 in our previous study (Supplementary Figure S2); therefore, we evaluated the expressions of SOD2 and GSH/GSSG (Figures 5J,K) to ascertain the antioxidative capability of BJD (Hass and Barnstable, 2021). In addition, we used the LCM-induced C2C12 myotube atrophy model for investigating the mechanisms of BJD alleviating muscle atrophy by affecting mitochondrial function *in vitro* as the C2C12 myoblast is a classical cell that is widely used for studying cancer-induced myotube atrophy. We found that BJD can promote myotube atrophy through mitochondrial function in the LCM-induced C2C12 myotube atrophy model which was consistent with our *in vivo* results (Figure 6). Collectively, the effectiveness of BJD in enhancing mitochondrial function both *in vivo* and *in vitro* was demonstrated by these findings.

However, the specific molecular mechanisms involved in mitochondrial function regulation remain unclear. In this study, the analysis of the KEGG signaling pathway demonstrated that the p53 signaling pathway, PI3K–Akt signaling pathway, or FoxO signaling pathway may be involved. FoxO was identified as the main transcription factor both in the ubiquitin–proteasome and the autophagy–lysosome which were two major proteolytic systems in the skeletal muscle during cancer cachexia (Sandri et al., 2004). FoxO-DNA binding–dependent transcription was necessary for muscle atrophy in cancer cachexia as mice with FoxO-DNA binding–dependent transcriptional blockade in the muscle were found to prevent muscle atrophy by inhibiting the increased mRNA levels of atrogin-1, MuRF-1, cathepsin L, and Bnip3, and increasing MyoD expression (Reed et al., 2012). Dysfunctional mitochondria, such as increased mitochondrial fission or deleted mitochondrial fusion, would be eliminated by mitophagy through mitochondrial cargo receptors including Bnip3, thus preserving mitochondrial function (Twig et al., 2008; Chen et al., 2010; Poole and Macleod, 2021). Therefore, BJD may regulate mitochondrial function by activating mitophagy via FoxO factors to prevent muscle atrophy in cancer cachexia. Despite the unclear mechanisms, p53 KO mice showed changes in mitochondrial function containing mitochondrial synthesis, mitochondrial dynamics, mitochondrial degradation, and complex IV assembly in the skeletal muscle (Saleem et al., 2015). In addition, we have previously found that the p38 MAPK/PGC1α signaling pathway was involved (Wang et al., 2020). Therefore, it still requires more investigations.

In conclusion, the results of our *in vivo* and *in vitro* studies suggest that BJD can prevent muscle atrophy by improving mitochondrial function in cancer cachexia. These findings also provide a scientific basis for treating cancer cachexia with traditional Chinese medicine.

## Data Availability

The original contributions presented in the study are publicly available. This data can be found here: [PRJNA865152].

## References

[B1] Argiles J. M. Busquets S. Stemmler B. Lopez-Soriano F. J. (2014). Cancer cachexia: understanding the molecular basis. Nat. Rev. Cancer 14 (11), 754–762. 10.1038/nrc3829 25291291

[B2] Argiles J. M. Fontes-Oliveira C. C. Toledo M. Lopez-Soriano F. J. Busquets S. (2014). Cachexia: a problem of energetic inefficiency. J. Cachexia Sarcopenia Muscle 5 (4), 279–286. 10.1007/s13539-014-0154-x 25118829 PMC4248416

[B3] Argiles J. M. Lopez-Soriano F. J. Busquets S. (2012). Counteracting inflammation: a promising therapy in cachexia. Crit. Rev. Oncog. 17 (3), 253–262. 10.1615/critrevoncog.v17.i3.30 22831156

[B4] Argiles J. M. Lopez-Soriano F. J. Busquets S. (2015). Muscle wasting in cancer: the role of mitochondria. Curr. Opin. Clin. Nutr. Metab. Care 18 (3), 221–225. 10.1097/MCO.0000000000000164 25769061

[B5] Aversa Z. Costelli P. Muscaritoli M. (2017). Cancer-induced muscle wasting: latest findings in prevention and treatment. Ther. Adv. Med. Oncol. 9 (5), 369–382. 10.1177/1758834017698643 28529552 PMC5424865

[B6] Baltgalvis K. A. Berger F. G. Pena M. M. Mark Davis J. White J. P. Carson J. A. (2010). Activity level, apoptosis, and development of cachexia in Apc(Min/+) mice. J. Appl. Physiol.(1985) 109 (4), 1155–1161. 10.1152/japplphysiol.00442.2010 20651218 PMC2963323

[B7] Baracos V. E. Martin L. Korc M. Guttridge D. C. Fearon K. C. H. (2018). Cancer-associated cachexia. Nat. Rev. Dis. Prim. 4, 17105. 10.1038/nrdp.2017.105 29345251

[B8] Baracos V. E. Mazurak V. C. Bhullar A. S. (2019). Cancer cachexia is defined by an ongoing loss of skeletal muscle mass. Ann. Palliat. Med. 8 (1), 3–12. 10.21037/apm.2018.12.01 30685982

[B9] Bodine S. C. Baehr L. M. (2014). Skeletal muscle atrophy and the E3 ubiquitin ligases MuRF1 and MAFbx/atrogin-1. Am. J. Physiol. Endocrinol. Metab. 307 (6), E469–E484. 10.1152/ajpendo.00204.2014 25096180 PMC4166716

[B10] Boengler K. Kosiol M. Mayr M. Schulz R. Rohrbach S. (2017). Mitochondria and ageing: role in heart, skeletal muscle and adipose tissue. J. Cachexia Sarcopenia Muscle 8 (3), 349–369. 10.1002/jcsm.12178 28432755 PMC5476857

[B11] Carson J. A. Hardee J. P. VanderVeen B. N. (2016). The emerging role of skeletal muscle oxidative metabolism as a biological target and cellular regulator of cancer-induced muscle wasting. Semin. Cell Dev. Biol. 54, 53–67. 10.1016/j.semcdb.2015.11.005 26593326 PMC4867246

[B12] Chan T. Y. (2009). Aconite poisoning. Clin. Toxicol. 47 (4), 279–285. 10.1080/15563650902904407 19514874

[B13] Chen H. Vermulst M. Wang Y. E. Chomyn A. Prolla T. A. McCaffery J. M. (2010). Mitochondrial fusion is required for mtDNA stability in skeletal muscle and tolerance of mtDNA mutations. Cell 141 (2), 280–289. 10.1016/j.cell.2010.02.026 20403324 PMC2876819

[B14] Constantinou C. Fontes de Oliveira C. C. Mintzopoulos D. Busquets S. He J. Kesarwani M. (2011). Nuclear magnetic resonance in conjunction with functional genomics suggests mitochondrial dysfunction in a murine model of cancer cachexia. Int. J. Mol. Med. 27 (1), 15–24. 10.3892/ijmm.2010.557 21069263 PMC3712618

[B15] de Castro G. S. Simoes E. Lima J. Ortiz-Silva M. Festuccia W. T. Tokeshi F. (2019). Human Cachexia Induces Changes in mitochondria, autophagy and apoptosis in the skeletal muscle. Cancers (Basel) 11 (9), E1264. 10.3390/cancers11091264 31466311 PMC6770124

[B16] Ding H. Jiang N. Liu H. Liu X. Liu D. Zhao F. (2010). Response of mitochondrial fusion and fission protein gene expression to exercise in rat skeletal muscle. Biochim. Biophys. Acta 1800 (3), 250–256. 10.1016/j.bbagen.2009.08.007 19716857

[B17] Fearon K. Strasser F. Anker S. D. Bosaeus I. Bruera E. Fainsinger R. L. (2011). Definition and classification of cancer cachexia: an international consensus. Lancet. Oncol. 12 (5), 489–495. 10.1016/S1470-2045(10)70218-7 21296615

[B18] Fermoselle C. Garcia-Arumi E. Puig-Vilanova E. Andreu A. L. Urtreger A. J. de Kier Joffe E. D. (2013). Mitochondrial dysfunction and therapeutic approaches in respiratory and limb muscles of cancer cachectic mice. Exp. Physiol. 98 (9), 1349–1365. 10.1113/expphysiol.2013.072496 23625954

[B19] Hass D. T. Barnstable C. J. (2021). Uncoupling proteins in the mitochondrial defense against oxidative stress. Prog. Retin. Eye Res. 83, 100941. 10.1016/j.preteyeres.2021.100941 33422637 PMC8263805

[B20] Huang S. Wang L. L. Xue N. N. Li C. Guo H. H. Ren T. K. (2019). Chlorogenic acid effectively treats cancers through induction of cancer cell differentiation. Theranostics 9 (23), 6745–6763. 10.7150/thno.34674 31660066 PMC6815948

[B21] Larsen S. Nielsen J. Hansen C. N. Nielsen L. B. Wibrand F. Stride N. (2012). Biomarkers of mitochondrial content in skeletal muscle of healthy young human subjects. J. Physiol. 590 (14), 3349–3360. 10.1113/jphysiol.2012.230185 22586215 PMC3459047

[B22] Ma Y. Liu Y. Teng L. Luo E. Liu D. Zhou F. (2021). Zi shen decoction Inhibits growth and metastasis of lung Cancer via regulating the AKT/GSK-3*β*/*β*-Catenin pathway. Oxid. Med. Cell. Longev. 2021, 6685282. 10.1155/2021/6685282 33777320 PMC7969097

[B23] Martin L. (2016). Diagnostic criteria for cancer cachexia: data versus dogma. Curr. Opin. Clin. Nutr. Metab. Care 19 (3), 188–198. 10.1097/MCO.0000000000000272 26945342

[B24] Neyroud D. Nosacka R. L. Judge A. R. Hepple R. T. (2019). Colon 26 adenocarcinoma (C26)-induced cancer cachexia impairs skeletal muscle mitochondrial function and content. J. Muscle Res. Cell Motil. 40 (1), 59–65. 10.1007/s10974-019-09510-4 30945134 PMC6692893

[B25] Penet M. F. Bhujwalla Z. M. (2015). Cancer cachexia, recent advances, and future directions. Cancer J. 21 (2), 117–122. 10.1097/PPO.0000000000000100 25815852 PMC4910156

[B26] Peris-Moreno D. Malige M. Claustre A. Armani A. Coudy-Gandilhon C. Deval C. (2021). UBE2L3, a partner of MuRF1/TRIM63, Is Involved in the degradation of myofibrillar actin and myosin. Cells 10 (8), 1974. 10.3390/cells10081974 34440743 PMC8392593

[B27] Pin F. Barreto R. Couch M. E. Bonetto A. O'Connell T. M. (2019). Cachexia induced by cancer and chemotherapy yield distinct perturbations to energy metabolism. J. Cachexia Sarcopenia Muscle 10 (1), 140–154. 10.1002/jcsm.12360 30680954 PMC6438345

[B28] Poole L. P. Macleod K. F. (2021). Mitophagy in tumorigenesis and metastasis. Cell. Mol. Life Sci. 78 (8), 3817–3851. 10.1007/s00018-021-03774-1 33580835 PMC8259496

[B29] Powers S. K. Wiggs M. P. Duarte J. A. Zergeroglu A. M. Demirel H. A. (2012). Mitochondrial signaling contributes to disuse muscle atrophy. Am. J. Physiol. Endocrinol. Metab. 303 (1), E31–E39. 10.1152/ajpendo.00609.2011 22395111 PMC3404565

[B30] Reed S. A. Sandesara P. B. Senf S. M. Judge A. R. (2012). Inhibition of FoxO transcriptional activity prevents muscle fiber atrophy during cachexia and induces hypertrophy. FASEB J. 26 (3), 987–1000. 10.1096/fj.11-189977 22102632 PMC3289501

[B31] Ren J. Sui H. Fang F. Li Q. Li B. (2019). The application of Apc(Min/+) mouse model in colorectal tumor researches. J. Cancer Res. Clin. Oncol. 145 (5), 1111–1122. 10.1007/s00432-019-02883-6 30887153 PMC11810213

[B32] Rohm M. Zeigerer A. Machado J. Herzig S. (2019). Energy metabolism in cachexia. EMBO Rep. 20 (4), e47258. 10.15252/embr.201847258 30890538 PMC6446208

[B33] Romanello V. Guadagnin E. Gomes L. Roder I. Sandri C. Petersen Y. (2010). Mitochondrial fission and remodelling contributes to muscle atrophy. EMBO J. 29 (10), 1774–1785. 10.1038/emboj.2010.60 20400940 PMC2876965

[B34] Sadeghi M. Keshavarz-Fathi M. Baracos V. Arends J. Mahmoudi M. Rezaei N. (2018). Cancer cachexia: Diagnosis, assessment, and treatment. Crit. Rev. Oncol. Hematol. 127, 91–104. 10.1016/j.critrevonc.2018.05.006 29891116

[B35] Saleem A. Iqbal S. Zhang Y. Hood D. A. (2015). Effect of p53 on mitochondrial morphology, import, and assembly in skeletal muscle. Am. J. Physiol. Cell Physiol. 308 (4), C319–C329. 10.1152/ajpcell.00253.2014 25472962 PMC4329426

[B36] Sandri M. (2016). Protein breakdown in cancer cachexia. Semin. Cell Dev. Biol. 54, 11–19. 10.1016/j.semcdb.2015.11.002 26564688

[B37] Sandri M. Sandri C. Gilbert A. Skurk C. Calabria E. Picard A. (2004). Foxo transcription factors induce the atrophy-related ubiquitin ligase atrogin-1 and cause skeletal muscle atrophy. Cell 117 (3), 399–412. 10.1016/s0092-8674(04)00400-3 15109499 PMC3619734

[B38] Schmidt S. F. Rohm M. Herzig S. Berriel Diaz M. (2018). Cancer Cachexia: More than skeletal muscle wasting. Trends Cancer 4 (12), 849–860. 10.1016/j.trecan.2018.10.001 30470306

[B39] Siddiqui J. A. Pothuraju R. Jain M. Batra S. K. Nasser M. W. (2020). Advances in cancer cachexia: Intersection between affected organs, mediators, and pharmacological interventions. Biochim. Biophys. Acta. Rev. Cancer 1873 (2), 188359. 10.1016/j.bbcan.2020.188359 32222610 PMC8132467

[B40] Siff T. Parajuli P. Razzaque M. S. Atfi A. (2021). Cancer-mediated muscle Cachexia: Etiology and Clinical management. Trends Endocrinol. Metab. 32 (6), 382–402. 10.1016/j.tem.2021.03.007 33888422 PMC8102392

[B41] So T. H. Chan S. K. Lee V. H. Chen B. Z. Kong F. M. Lao L. X. (2019). Chinese medicine in Cancer treatment - how is it practised in the east and the west? Clin. Oncol. 31 (8), 578–588. 10.1016/j.clon.2019.05.016 31178347

[B42] Suzuki T. Von Haehling S. Springer J. (2020). Promising models for cancer-induced cachexia drug discovery. Expert Opin. Drug Discov. 15 (5), 627–637. 10.1080/17460441.2020.1724954 32050816

[B43] Twig G. Elorza A. Molina A. J. Mohamed H. Wikstrom J. D. Walzer G. (2008). Fission and selective fusion govern mitochondrial segregation and elimination by autophagy. EMBO J. 27 (2), 433–446. 10.1038/sj.emboj.7601963 18200046 PMC2234339

[B44] van der Ende M. Grefte S. Plas R. Meijerink J. Witkamp R. F. Keijer J. (2018). Mitochondrial dynamics in cancer-induced cachexia. Biochim. Biophys. Acta. Rev. Cancer 1870 (2), 137–150. 10.1016/j.bbcan.2018.07.008 30059724

[B45] Wallace D. C. (2005). A mitochondrial paradigm of metabolic and degenerative diseases, aging, and cancer: a dawn for evolutionary medicine. Annu. Rev. Genet. 39, 359–407. 10.1146/annurev.genet.39.110304.095751 16285865 PMC2821041

[B46] Wang D. Chen W. Bi Q. Zong X. Ruan J. Yin X. (2020). Baoyuan jiedu decoction alleviates Cancer-Induced myotube Atrophy by regulating mitochondrial dynamics through p38 MAPK/PGC-1α signaling pathway. Front. Oncol. 10, 523577. 10.3389/fonc.2020.523577 33102208 PMC7556243

[B47] Wang M. Yin X. Wang D. Ji X. (2021). Clinical observation of Baoyuan Jiedu decoction on lung cancer related fatigue. Clin. J. Chin. Med. 13 (03), 83–86.

[B48] Xu R. Wu J. Zhang X. Zou X. Li C. Wang H. (2020). Modified Bu-zhong-yi-qi decoction synergies with 5 fluorouracile to inhibits gastric cancer progress via PD-1/PD- L1-dependent T cell immunization. Pharmacol. Res. 152, 104623. 10.1016/j.phrs.2019.104623 31899315

[B49] Yan Z. Lira V. A. Greene N. P. (2012). Exercise training-induced regulation of mitochondrial quality. Exerc. Sport Sci. Rev. 40 (3), 159–164. 10.1097/JES.0b013e3182575599 22732425 PMC3384482

[B50] Yang G. W. Jiang J. S. Lu W. Q. (2015). Ferulic acid exerts anti-angiogenic and anti-tumor activity by targeting fibroblast growth factor receptor 1-mediated angiogenesis. Int. J. Mol. Sci. 16 (10), 24011–24031. 10.3390/ijms161024011 26473837 PMC4632735

[B51] Zdunska K. Dana A. Kolodziejczak A. Rotsztejn H. (2018). Antioxidant properties of ferulic acid and Its possible application. Skin. Pharmacol. Physiol. 31 (6), 332–336. 10.1159/000491755 30235459

[B52] Zhang H. Zong X. Deng T. ZHao R. Ji X. (2018). Mechanisms of Baoyuan Jiedu decoction in the intervention of carcinogenic muscular atrophy through inhibiting cytokins-ubiquitin-proteasome pathway. J. Beijing Univ. Trad. ChinMed 41, 642–647.

[B53] Zhang Y. Han X. Ouyang B. Wu Z. Yu H. Wang Y. (2017). Chinese herbal medicine baoyuan jiedu decoction Inhibited muscle atrophy of Cancer Cachexia through atrogin-l and MuRF-1. Evid. Based. Complement. Altern. Med. 2017, 6268378. 10.1155/2017/6268378 PMC5329682 28286533

[B54] Zong X. Zhang Y. Zhang H. Ji X. (2019). Mechanism of baoyuan jiedu decoction in alleviating muscle atrophy in Apcmin/+ cachexia mice. Chin. J. Exp. Trad. Med. Formul. 25, 19–24.

