# Supplementary material for: Baoyuan Jiedu decoction alleviating cancer cachexia–Induced muscle atrophy by regulating muscle mitochondrial function in Apc Min/+ mice
Source: Front Pharmacol. 2022 Aug 19;13:914597. doi: 10.3389/fphar.2022.914597 (PMC9437209; doi:10.3389/fphar.2022.914597)

Supplemental information

Baoyuan Jiedu Decoction Alleviating Muscle Atrophy in Cancer-associated Cachexia by Regulating Muscle Mitochondrial Function in Apc^Min/+^ Mice

Beiying Zhang^1*^, Qianyu Bi^1*^, Shengqi Huang^1*^, Siyuan Lv^1*^, Xin Zong^3^, MengRan Wang^4^, Xuming Ji^1,2^

*^1^School of Basic Medical Science, Zhejiang Chinese Medical University, Zhejiang, China*

*^2^Academy of Chinese Medical Science, Zhejiang Chinese Medical University, Zhejiang, China*

*^3^Weifang Nursing Vocational College, Shandong, China*

*^4^Department of Pediatrics, Affiliated Hospital of Shandong University of Traditional Chinese Medicine, Shandong, China*

Correspondence should be addressed to Xuming Ji; jixuming724@163.com

*These authors have contributed equally to this work


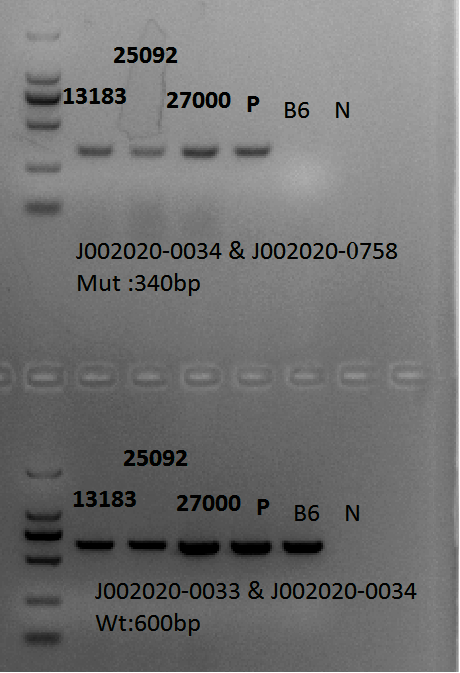


Supplemental Figure1. qPCR of the determination of *Apc^Min/+^* Mice

Note: the primers are shown in Supplemental Table4.


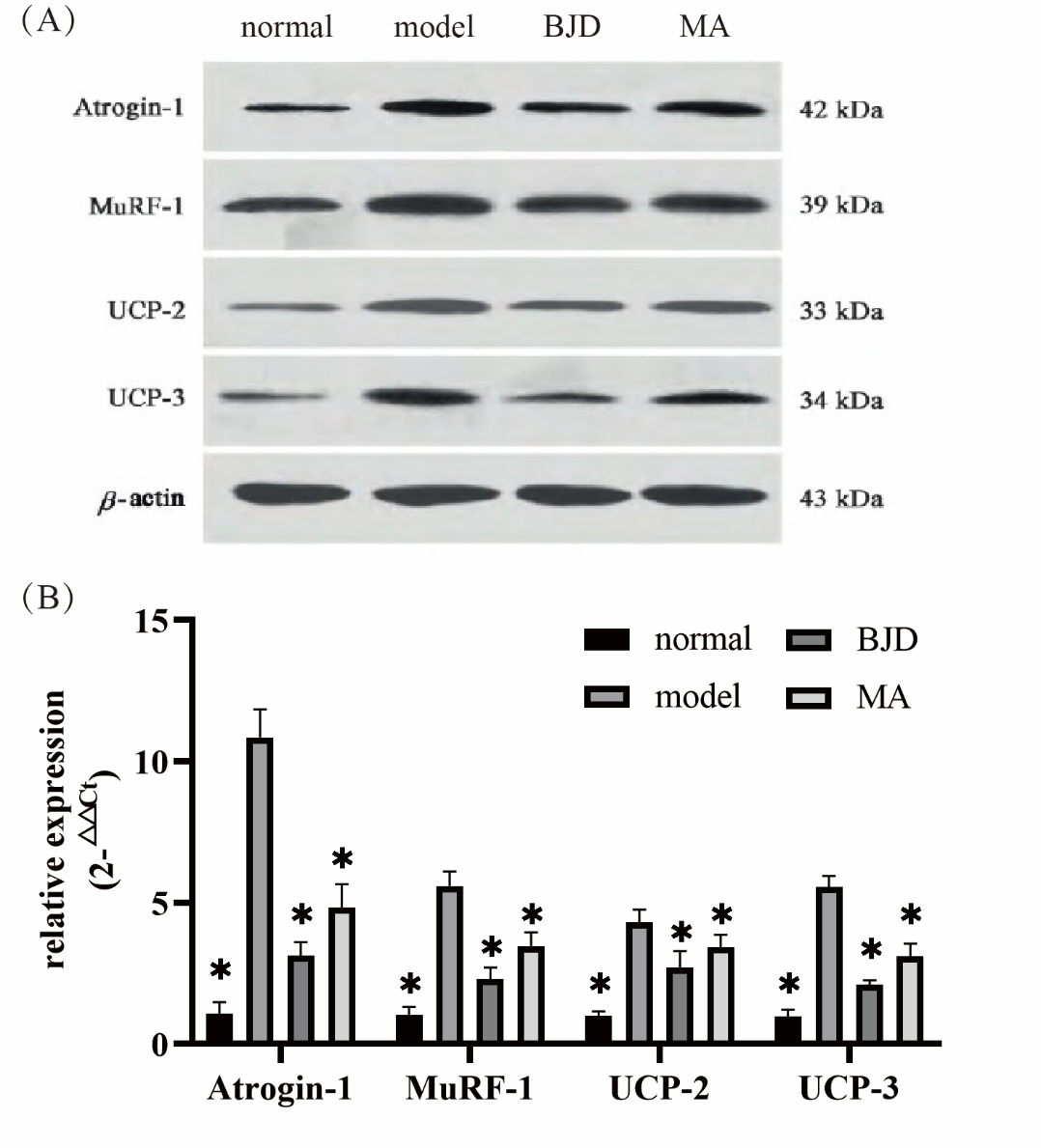


Supplemental Figure2. Protein(A) and mRNA(B) expressions of atrogin-1, MuRF-1, UCP2, and UCP3 in *Apc^Min/+^* Mice

Note: this picture was from our previous study. (Compared with model group, **P*<0.05)

Supplemental Table1. The information on HPLC conditions

|  | mobile phase | wave length | colomn temperature | flow rate | sample injection volume |
| --- | --- | --- | --- | --- | --- |
| chlorogenic acid | acetonitrile-0.1%phosphoric acid: 9:91 | 325nm | 40℃ | 1 mL/min | 20uL |
| ferulic acid | acetonitrile-0.1%phosphoric acid: 9:91 | 315nm | 20℃ | 1 mL/min | 20uL |
| aconitine | acetonitrile-0.05%phosphoric acid water 0-25min, acetonitrile 30-35% | 230nm | 35℃ | 1 mL/min | 20uL |

Supplemental Table2. The information on standard samples

| standard sample(μg/mL) | mass of standard sample(mg) | methanol |
| --- | --- | --- |
| 20 μg/mL chlorogenic acid | 0.2 | 80% methanol |
| 20 μg/mL ferulic acid | 0.2 | 70% methanol |
| 50 μg/mL aconitine | 0.5 | 70% methanol |

Note: chlorogenic acid (Shanghai yuanye Bio-Technology Co., China, Cat No: B20782); ferulic acid (Shanghai yuanye Bio-Technology Co., China, Cat No: B20007); aconitine (Chem Faces, China, Cat No: CFN99915).

Supplemental Table3. Primers

| gene | primers |
| --- | --- |
| MURF-1 | forward 5'-GAGAACAGTATGGGGTCA-3', |
|  | reverse 5'-TAATAAAGTCTTGGGGTG-3' |
| Atrogin-1 | forward 5'-GCCACCTTCCTCTTGAGT-3', |
|  | reverse 5'-CCTTGTTCTGTCTTCCCC-3' |
| NRF-1 | forward 5'-GCACAGAAGAGCAAAAG-3', |
|  | reverse 5'-CGAAAGCATACAGAAGG-3' |
| NRF-2 | forward 5'-AGAAAAGGGAGAAAACGACA-3', |
|  | reverse 5'-TTCACTGGGAGAGTAAGGCT-3' |
| TFAM | forward 5'-GTGGGGCGTGCTAAGAAC-3', |
|  | reverse 5'-GCTGACAGGCGAGGGTAT-3' |
| COXIV | forward 5'-TGAGATGAACAAGGGCACCA-3', |
|  | reverse 5'-CACCCAGTCACGATCAAAGG-3' |
| Cyt C | forward 5'-ACCCTGATGGAGTATTTG-3', |
|  | reverse 5'-GCTATTAGTCTGCCCTTTC-3' |
| Mfn1 | forward 5'-CTTCTAACCCAGCAGCCCC-3', |
|  | reverse 5'-TGTTTTCCAAATCACGCCC-3' |
| Mfn2 | forward 5'-ACGGAGGAAGTGGAAAGGCA-3', |
|  | reverse 5'-TGAGGACAACTGGGGATGGG-3' |
| Fis1 | forward 5'-GAGCCCCAGAACAACCAGG-3', |
|  | reverse 5'-CAGTCCAATGAGTCCAGCC-3' |
| mtDNA | forward 5'-CGATAATCCCCGCTCTACC3-3', |
|  | reverse 5'-AGCCCATTTCTTCCCATTTC-3' |
| MyoD | Forward 5'-CAGCAAGATACAGAATGGT-3', |
|  | reverse 5'-TGAGAGACAGGAGATGACA-3' |
| β-actin | forward 5'-CCTAGACTTCGAGCAAGAGA-3', |
|  | reverse 5'-GGAAGGAAGGCTGGAAGA-3' |

Supplemental Table4. Primers

| gene | primers |
| --- | --- |
| Wild | forward 5'-GCCATCCCTTCACGTTAG-3', |
|  | reverse 5'-TTCCACTTTGGGCATAAGGC-3' |
| Apc | forward 5'-TTCCACTTTGGGCATAAGGC-3', |
|  | reverse 5'-TTCTGAGAAAGACAGAAGTTA-3' |


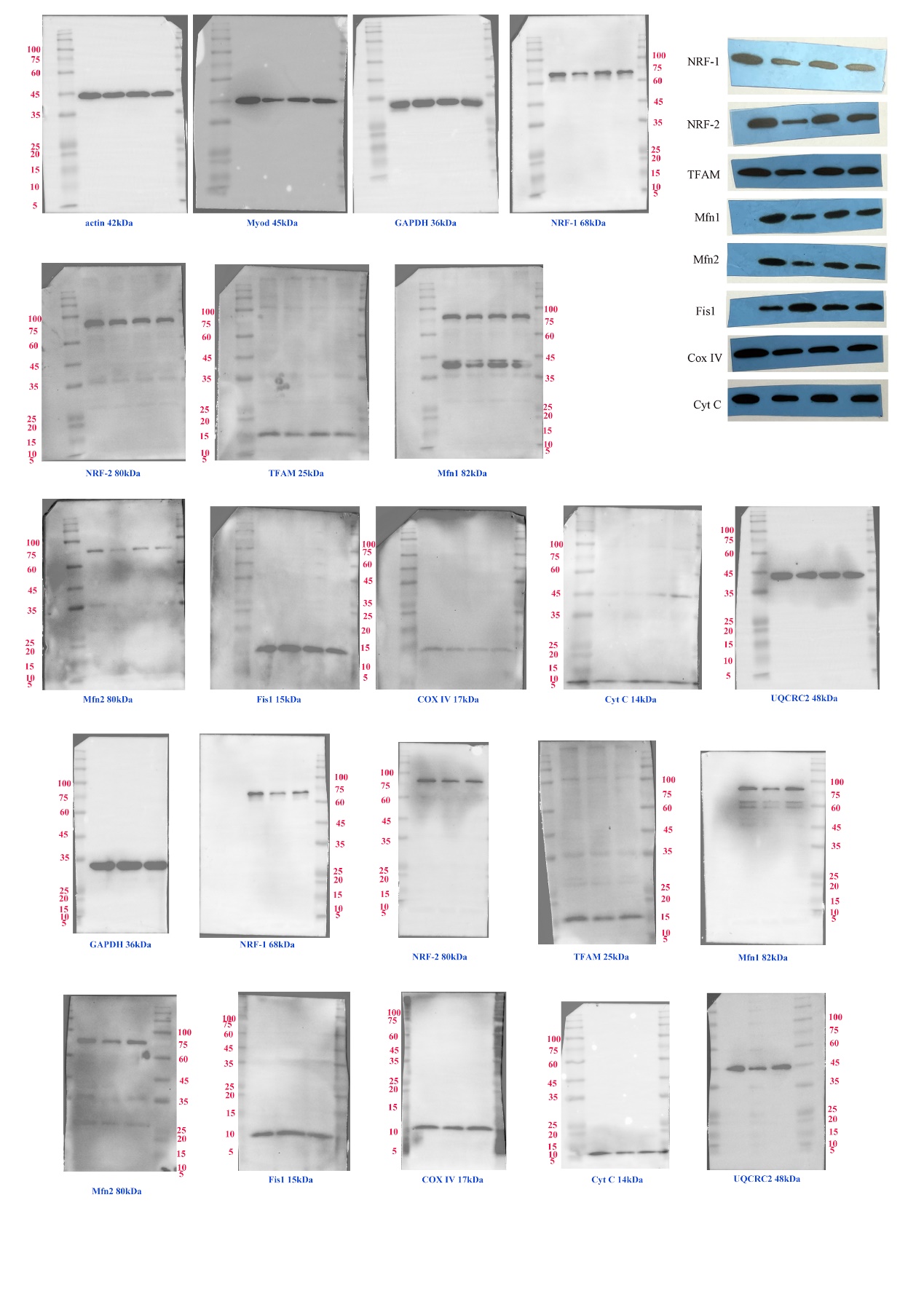

Supplement: Supplementary file 1 [file DataSheet1.docx]
